# Stigma reduction as quality health care: A qualitative study of structural level mechanisms to address stigma

**DOI:** 10.1371/journal.pone.0357633

**Published:** 2026-09-08

**Authors:** Carla Treloar, Emily Lenton, Amy Kirwan, Caitlin Douglass, Gemma Nourse, Adrian Farrugia, Kate Seear, Caroline Watts, Mark Stoove, Elena Cama

**Affiliations:** 1 Centre for Social Research in Health, UNSW Sydney, Sydney, Australia; 2 Australian Research Centre in Sex Health and Society, La Trobe University, Melbourne, Australia; 3 Burnet Institute, Melbourne, Australia; 4 School of Public Health and Preventive Medicine, Monash University, Melbourne, Australia; 5 Melbourne School of Population and Global Health, University of Melbourne, Melbourne, Australia; 6 National Drug Research Institute, Curtin University, Melbourne, Australia; 7 Deakin Law School, Deakin University, Melbourne, Australia; 8 Kirby Institute, UNSW Sydney, Sydney, Australia; University of Warwick, UNITED KINGDOM OF GREAT BRITAIN AND NORTHERN IRELAND

## Abstract

The impacts of stigma on health are well-known. Most interventions to reduce stigma focus on education or skills building of health workers and less on organisational or structural approaches. Responding to ongoing efforts to increase the quality of health care, we explore data from semi-structured interviews with 20 key stakeholders to identify and examine stigma reduction opportunities that align with existing practices, policies and structures for strengthening quality in the Australian healthcare system. Analysis focused on the “outer setting” constructs of the Consolidated Framework for Implementation Research to identify and categorise mechanisms that linked to or were determined by governing processes of the (“outer setting”). The necessity to be accredited against national standards was positioned as an over-arching driver of what health systems do and what gets the attention of leaders and executives, and can subsequently impact allocation of resources, including for stigma reduction. Participants’ accounts also raise the possibility of using other more general industrial relations laws to bring greater attention to stigma reduction. Innovative approaches to stigma reduction using macro, structural tools provide a promising opportunity to trial.

## Introduction

The negative impacts of stigma on healthcare access and outcomes are well-documented [1]. Interventions to address stigma in healthcare have typically focused on providing health practitioners with information and skills, and contact with particular stigmatised groups [2]. Interventions targeted at organisational and structural levels of stigma, focusing on stigma within institutions and legislative and policy environments, have received less attention [3,4]. Other authors have drawn attention to the need for organisational and structural level collusion with quality care initiatives for effective and efficient ways to reduce stigma [5]. That is, embedding aims to reduce stigma within the “already well accepted processes and priorities for health-care providers and organisations” to improve quality care [6] (p864). In this paper, we explore what key stakeholders identify to be stigma reduction opportunities that align with existing practices, policies and structures for strengthening quality in the Australian healthcare system.

Low quality healthcare produces “staggering” financial and social costs, and providing high quality care is a priority for all health systems globally to deliver best outcomes [7]. A Lancet Commission noted that high quality care “should be in the DNA of all health systems” but acknowledges available care is “worst” for marginalised groups, including those who are stigmatised [8]. Despite a range of legislation, policies and practices that have been developed over this century to promote higher quality healthcare, stigma continues to occur and generates ongoing harms for individuals and communities, and preventable costs to the health system and society [1,4].

Investigations of mechanisms to address stigma need to be built on a clear operationalised definition. Stigma can be understood as a social process, whereby a person or group of people is devalued or excluded on the basis of actual or perceived characteristics that are viewed negatively by a broader social group [9]. Link and Phelan’s well-established conceptualisation of stigma provides five inter-related components, namely: labelling, stereotyping, separation, and status loss, in the context of power differentials [10]. They argue that the status loss inherent to stigma relies on and reproduces power imbalances. While stigma can be conceptualised as operating over multiple levels (individual, interpersonal, organisational/structural), efforts to address stigma generally address professional practice (interpersonal level) and pay less attention to structural drivers of stigma in healthcare [1]. Structural stigma can be conceptualised as embedded in, and reproduced by, institutional policies, frameworks, laws, and regulations [11,12]. Strategies to reduce stigma should then logically “look up” [13] to identify and target structural forces that can sustain or mitigate stigma.

Attention to structural levers is also important as research over decades has shown that stigma and its effects are not readily or enduringly dismantled by commonly employed education or information interventions [2,14,15]. While such programs are well-intended, their impact is often localised or not able to be sustained because they are not integrated into normalised operations as “sustained, integrated” approaches at structural levels [16]

A close examination of structural mechanisms for stigma reduction also directs attention to analyses of power within healthcare systems. Orienting to power and institutional processes in this way places the onus to act on the organisation, rather than the individual, can challenge the power relations that sustain it [13–15].

The available literature identifies a few mechanisms useful for this analysis. Investigating structural stigma and quality care in the healthcare experiences of people living with mental health and substance issues, Livingston (2020) draws attention to the importance of enhancing and enforcing protections for people seeking healthcare. Key to consumer protections are accreditation processes by which health service organisations and health workers are assessed on their competency with, and implementation of, safety and quality standards [17,18]. With particular relevance to stigma reduction, “accreditation organizations have a role to play in ensuring that health-care organizations establish and implement policies that respect people’s rights, guard against coercive practices, reduce inequities and disparities, provide access to quality care, and promote the participation of people with lived experience” (p21) [11]. Continuous quality improvement processes are a key activity of healthcare organisations that can provide evidence of action on adherence to quality standards in accreditation assessments [19]. Other authors have also emphasised the possibilities of stigma-focused quality improvement processes to improve stigma literacy and action in existing health system processes [6], with further research needed to support operationalisation of this emergent area [20–22]. There is also need for a focused investigation of this and other possible existing tools of health system governance that can be used in a “strategic collusion” of stigma reduction with the imperative for high quality care [23].

Efforts to examine how stigma can be addressed using structural mechanisms has the potential to benefit many people. The experience of stigma is highly prevalent as many health conditions, identities and practices are stigmatised. In a sample of the US population, Pachankis and colleagues identified 93 stigmatised conditions and estimated that 90% of the population live with at least one [24]. We suggest that it is not feasible for health systems to design and implement independent stigma reduction interventions specific to each potentially stigmatised characteristic [25]. A more feasible approach would identify systems-level mechanisms to which stigma reduction can be written in [26] to drive collective and system-wide action. While ambitious, this approach is more likely to benefit the large number and range of stigmatised conditions, identities and practices.

In this study, we examined the perspectives of key stakeholders in the blood-borne virus and sexually transmissible (BBV/STI) sector in relation to the systems-level processes that could be adapted and implemented as stigma reduction interventions. We chose this sector as a focus noting the salience in the BBV/STI sector of the root causes of stigma (concealability, course, disruptiveness, aesthetics, origin, and peril) [27]. That is, BBVs such as HIV have historically been stigmatised both due to perceptions of peril around the virus itself (such as fear of infection, misinformation) and negative attitudes (such as disgust, origin and blame, aesthetics) towards potential transmission routes (e.g., injecting drug use, unprotected sex) [28]. Features of the health system that were identified as useful for BBVs/STIs could be explored for their applicability and adaptability for other conditions, identities, and practices.

## Methods

This paper draws on qualitative interviews with 20 key stakeholders who have extensive experience working in the BBV/STI sector. This analysis was conducted as part of a larger study aiming to design and implement a stigma reduction approach that moves beyond identity- or condition-specific siloes [25]. While this research has identified a strong investment in reducing stigma among professionals working in BBV/STI [29] and examined some potential strategies for service design and delivery [30], more needs to be known about the feasibility of these structural mechanisms for addressing the stigmatisation of BBV/STIs and beyond.

Key stakeholders were purposively identified by the research team who have extensive experience and professional networks in the sector. The team sought to ensure a spread of expertise in health and community settings, representing Australian jurisdictions, a balance of experience and knowledge across viral hepatitis, HIV and other STIs, and experience working in urban, outer urban and regional contexts. The stakeholders held current or previous roles in policy, management and service provision across primary, community and tertiary settings, in peer-led and identified roles and organisations, professional associations and research.

Invitations to participate were extended by email directly to individuals across the selection criteria identified by the research team (beginning 13 December 2022 and ceased on 26 November 2023). All stakeholders agreed to participate, however due to travel and international time differences, one interview did not transpire, and a suitable representative from the service was interviewed. Stakeholders accepting the invitation were scheduled for an interview at their convenience. Interviews were conducted by EL and GN. Interviews were guided by a semi-structured schedule developed to identify individual, service and structure-levels in which BBV/STI-related stigma in Australian healthcare unfolds, as well as opportunities for reform. Our understanding of structural stigma in the BBV/STI sector is informed by our previous research speaking to governance processes and accreditation [12] as well as frameworks for understanding and responding to structural stigma in mental health [11]. In alignment with the aims of the project, stakeholders were asked to reflect on and respond to the concept of a ‘universal precautions’ approach to stigma reduction. This included topics such as the acceptability of the term, the feasibility of developing, designing and implementing a universal framework to stigma reduction and measuring change. They were asked to identify existing structures, policies, procedures, and frameworks where there is capacity to embed stigma reduction interventions.

All interviews were conducted via Zoom or Teams with the video function turned on and were between 50–100 minutes in length. Interview recordings were transcribed, checked for accuracy, and deidentified by the interviewers (EL, GN). Initial coding of the transcripts identified participant accounts of organisational and structural elements important to stigma. A second stage of analysis used a pragmatic approach [31] to note existing structural levers identified by participants that could be implemented for stigma reduction initiatives in healthcare. Analysis was guided by the Consolidated Framework for Implementation Research (CFIR) [32] which is a highly cited and widely used conceptual tool for explaining barriers and facilitators to implementation effectiveness [32]. The use of CFIR prior to implementation of a program is uncommon [33] but useful to identify factors that can inform development and roll-out of future interventions [34]. While activities or mechanisms to support stigma reduction might operate within a healthcare organisation (the “inner setting” as considered by CFIR), we looked for mechanisms that linked to or were determined governing processes (“outer setting”), such as those emerging from the state more broadly. Accreditation to quality standards is one example of a mechanism in the “outer setting” that has been highlighted in the literature on responses to structural stigma [11]. We used constructs of the CFIR “outer setting” to categorise other activities and their governance processes that were identified or alluded to by participants (see Table 1 for definitions). In inductive analysis, we examined how participants perceived the utility and application of these mechanisms to support stigma reduction interventions, particularly identifying where participants were critical or sceptical of these aspects of the “outer setting”.

**Table 1:** Outer setting construct (and sub-domains) of the Consolidated Framework for Implementation Research (CFIR) [32].

*Outer Setting:* The setting in which the Inner Setting exists, e.g., hospital system, school district, state. There may be multiple Outer Settings and/or multiple levels within the Outer Setting (e.g., community, system, state).	
A. Critical Incidents	Large-scale and/or unanticipated events disrupt implementation and/or delivery of the innovation.
B. Local Attitudes	Sociocultural values (e.g., shared responsibility in helping recipients) and beliefs (e.g., convictions about the worthiness of recipients) encourage the Outer Setting to support implementation and/or delivery of the innovation.
C. Local Conditions	Economic, environmental, political, and/or technological conditions enable the Outer Setting to support implementation and/or delivery of the innovation.
D. Partnerships & Connections	The Inner Setting is networked with external entities, including referral networks, academic affiliations, and professional organization networks.
E. Policies & Laws	Legislation, regulations, professional group guidelines and recommendations, or accreditation standards support implementation and/or delivery of the innovation.
F. Financing	Funding from external entities (e.g., grants, reimbursement) is available to implement and/or deliver the innovation.
G. External Pressure	External pressures drive implementation and/or delivery of the innovation. *Use this construct to capture themes related to External Pressures that are not included in the subconstructs below.*
1. Societal Pressure	Mass media campaigns, advocacy groups, or social movements or protests drive implementation and/or delivery of the innovation.
2. Market Pressure	Competing with and/or imitating peer entities drives implementation and/or delivery of the innovation.
3. Performance-Measurement Pressure	Quality or benchmarking metrics or established service goals drive implementation and/or delivery of the innovation.

The study was approved by University of New South Wales, following which it was ratified by the La Trobe University Human Research Ethics Committee (HC220669). All participants provided written consent.

## Results

### Demographic characteristics of the sample

Interviews were conducted with 20 stakeholders located in the Australian states of Victoria (n = 6), New South Wales (n = 7), South Australia (n = 3) and one each in Queensland, Western Australia, the Australian Capital Territory, and the Northern Territory. Participants were employed primarily in roles within their state (n = 14) as well as national (n = 5) and internationally based roles (n = 1). Participants had extensive experience ranging from 7–47 years in the BBV/STI sector and at the time of interview worked across direct service provision (n = 6), community and peer advocacy (n = 4), policy (n = 5), professional associations (n = 2), and in university research or teaching roles (n = 3).

### Accreditation to national safety and quality standards

Accreditation processes featured in participant narratives about governance processes that were or could be aligned with stigma reduction goals. The “outer setting” constructs of *Policies and Laws* as well as *Performance Measurement Pressure* were reflected in participant comments about the possibility of driving change by embedding stigma into standards governed by the Australian Commission on Safety and Quality Health Care (the national healthcare standards agency for all health services, general practices, pathology and medical imaging providers in Australia), and the associated accreditation processes required for healthcare organisations and health workers. Numerous participants had direct experience of working with the standards, including gathering required documentation, sitting on working and advisory committees, teaching to undergraduate students and developing new standards. The standards were described as ‘the bible’, providing benchmarks of ‘best practice’ (P1, research, education/training), and accreditation was understood as a ‘rigorous’, ‘formal and legal process’ (P18, peak body). While standards refer to operations of health services, participants noted a “cascade effect” (P1, research, education/training) that accreditation affords, whereby these standards then become embedded within tertiary education, underpinning organisational codes of conduct, policies such as diversity and inclusion, and quality improvement processes, including client/patient evaluations in practice.

The current quality standards for Australian healthcare do not include a component or standard directly addressing stigma in healthcare. Participants identified that two of the eight current standards are partly relevant to stigma; ‘partnering with consumers’ and ‘communicating for safety’ standards. Typically, participants described that health organisations form committees or working groups to respond to accreditation requirements against these and other standards. Processes to evaluate compliance with these standards were reported as focused on examination of local data (such as patient or staff surveys, clinical data regarding adverse events or near misses, and complaints data). Some participants suggested other strategies to adapt accreditation processes that have the capacity to capture and identify occasions of stigma and ensure meaningful engagement with consumers. Here, P7 (tertiary, management) shares an approach they suggest could “get to the heart of a service”:

I am big fan of the idea of mystery shoppers […] I sometimes wonder you know to really get to the heart of a service and what it’s doing and what its attitudes are [if it could be useful to go] through […] simulation exercises, those hypotheticals with staff, or using the mystery shopper concept.

The CFIR “outer setting” construct of *Partners and Connections* aligns closely with a component of the current quality and safety standards: ‘partnering with consumers’. This component recognises the importance of working with consumers in the planning and delivery of healthcare and providing clear communication to minimise risks of harm [35]. This standard provides a significant point of congruence for stigma reduction initiatives, as direct involvement of people affected by stigma is best practice for stigma reduction efforts [36] and in quality improvement processes [37]. Participants noted that this standard provided particular opportunities and challenges to advance stigma reduction initiatives. P7 (tertiary, management) noted this standard was “one of the more useful ones […] when they ask about services and stakeholder engagement, and whether or not they do interact with their client groups and with other marginalised communities”. Participants suggested that institutional structures such as committees and working groups for each standard that encouraged “authentic engagement” with consumers (P13, tertiary, higher education) can shift stereotypes and perceptions of people who are marginalised and “enable change” (P13). Participants also identified challenges and limitations to this process, including the possibility that the involvement of communities might falter without strong leadership and investment of resources. In a large and resource-constrained health service, consumer involvement could be reduced or minimised, with capacity for only one representative among a larger contingent of staff, making it difficult for consumer representatives to contribute and effectively silencing the input from people living with many other stigmatised conditions, identities and practices.

The CFIR “outer setting” construct of *Local Attitudes*, referring to sociocultural values and beliefs, is useful in examining the more critical perspective of some participants who indicated that accreditation processes were ineffective to support real change on stigma. In these instances, even rigorous accreditation exercises were perceived as simply a ‘tick and flick’ exercise, unable to ‘change attitudes’ or get ‘to the heart of a service’ (P7, tertiary setting, management) or change broader perceptions regarding the relative worthiness of care recipients. This perception was illustrated in participants’ comments about the burden and ineffectiveness of accreditation processes especially for services and institutions subject to a number of different accreditation standards that may be resource intensive and complex.

### Other governance structures useful for alignment with stigma reduction

Accreditation requirements (*Policies and Laws* within the “outer setting”) were seen to have the potential to drive the management team of a health service to focus on stigma reduction initiatives in order to fulfil certain standards or requirements. Suggestions provided by participants mapped to other constructs of the CFIR “outer setting”. In particular, the *External Pressure* of performance targets for health service leadership was seen as fruitful for influencing priorities for stigma reduction initiatives achieved with “not a lot of effort” (P14, program design, lived experience). These measures might be outside of quality standards and sit within, for example, performance measures set by local governance (jurisdictional governments or the governing boards of health districts) to drive efforts in line with strategic priorities as Participant 14 (program design, lived experience) explained.

Chief executives are given performance agreements with health departments which say, “you will do these things”, and it’s amazing what happens when that happens, you get a bit of focus on it. So, I think there are those channels which [for] not a lot of effort from […] a top-of-the-tree policy perspective setting the expectation, I don’t think that should be that difficult.

As health professionals of long-standing experience, participants identified a number of other *Policies and Laws* that influenced and governed their practice. These included codes of conduct that health workers are required to sign on obtaining employment in health organisations, and which often form parts of employment contracts. Competency standards and professional practice standards were also noted as guidelines against which health workers were held accountable. However, participants provided critique of the “retrospective” (P17, Professional association, policy) nature of these accountabilities which are activated only if a complaint is lodged about the practitioner or organisation to compliance organisations or regulatory bodies. Even though complaints could be seen as a *Critical Incident* (CFIR “outer setting” construct), the retrospective application of these professional standards on the basis of complaints meant that they were passive tools for accountability and rarely used. Despite quality standards indicating that complaints should be a key piece of data for quality improvement, participants understood that there are numerous barriers to making complaints. Here P18 sees the lack of awareness of these mechanisms as a structural concern:

I was astonished to hear that people didn’t know about […] health complaints commission you know that there were other mechanisms and I thought “well, why don’t they, they should really know about that upfront, like that’s a problem with the system if you don’t know what to do and how to do it”. (P18, Professional association, policy)

Within the workplace, *Performance Measurement Pressure* (CFIR “outer setting” construct) for individual workers was also recognised as a possible means to support stigma reduction initiatives. Some participants saw a “lot of overlap” (P7, tertiary, management) between stigma reduction initiatives within the workplace and legal responsibilities for the organisation to create “respectful workplaces” (P7, tertiary, management). For example, P7 (tertiary, management) described that a “whole ecosystem” action against “bullying and disrespect” has the effect of “[creating] respect between our colleagues and then we also create respect between our clients and providers”. Participants noted that regular processes of institutional management and workforce development could be adapted to reflect, endorse and measure codes of conduct and core values which are taken “seriously enough” as a “disciplinary issue” (P15, government, policy). These processes included interview questions, on-boarding protocols, mandatory training and performance appraisals.

[in performance appraisals] there is nothing there about “what do you feel around judgement and stigmatisation?” Maybe that could be woven into a general annual performance appraisal, because that would then give the manager a right then to be able to address it then and there on the spot; “Well actually you have written that you think you are really good and in fact I am hearing patients of the service or other colleagues that behind patient’s backs you have actually been quite, you know, discriminating”. (P16, tertiary, health promotion)

## Discussion

In this paper, we examined the perceptions of experienced health workers in relation to possibilities that structural-level governing mechanisms that could be “put to use” and implemented to support stigma reduction efforts in healthcare. Participant accounts support the argument for developing and testing a strategic collusion, a systematic and deliberate bringing together, of quality precepts and stigma reduction achieved via structures and processes of the “outer setting” of health organisations to which organisations are legally and financially beholden. Participants also note that while there may be possibility of using these structural supports, there is enduring challenge for genuine implementation beyond “tick and flick” pro forma responses. In this paper, these structural levers aligned with the goal of stigma reduction were characterised using a number of CFIR constructs of the “outer setting”, particularly *Laws and Policies* and *Performance Measures*.

The necessity to be accredited against national standards was positioned as an over-arching driver of what health systems do and what gets the attention of leaders and executives, and can subsequently impact allocation of resources. Participant discussions of accreditation focused on its potential usefulness to focus the attention of health executives to the matter as an *External Pressure*. While the current standards for general healthcare in Australia do not make reference to stigma, changes to the standard for the provision of aged care in Australia (implemented late 2025) have included reference to discrimination, such that: “The provider must deliver funded aged care services to individuals in a way that is free from all forms of discrimination, abuse and neglect, treats individuals with dignity and respect, and respects the personal privacy of individuals” [38] (p10). This is a promising step forward and might be useful to direct providers’ attention and resources to additional requirements for policies, processes and staff training. While age and some aspects of health status are protected attributes in anti-discrimination law, not all concerns of the public in relation to stigma are protected, and stigma may be seen as outside of these standards as it does not meet the legal definition of discrimination [39]. The specification of stigma (and not only discrimination) is important for any re-writing of quality standards.

Participants’ accounts also raise the possibility of using other more general industrial relations laws to bring greater attention to stigma reduction. References to workplace responses to “bullying” and the promotion of “respectful workplaces” align with recent laws placing positive responsibilities on employers to avoid psychosocial hazards (anything that could cause psychological harm, e.g., harm someone’s mental health) [40]. These laws could also be used to promote stigma reduction in relation to efforts to address witnessed stigma through bystander responses [41] or positive mechanisms for staff to report or seek remedy on behalf of patients, such as complaints mechanisms.

Complaints mechanisms were mentioned less frequently by participants than quality standards, although the two processes are inherently tied. The current standards indicate that complaints mechanisms should inform improvement in quality of services [35], but the broader literature indicate that complaints data are underutilised as a source of data, analysis, and action for quality improvement [42,43]. Complaints may also be underutilised by health consumers because of lack of knowledge (as participants suggest), but also because the complaints process is onerous and upholds the power imbalance between consumers and health services and can reinforce stereotypes [15,44]; consumers may want to complain about services but may be reliant on them for ongoing care [45]. In CFIR terms, the low use of complaints mechanisms relates to the relative absence of the “outer setting” construct of *Societal Pressure* as a driver of stigma reduction initiatives. In interpreting this, we refer back to Fraser’s conceptualisation of stigma and power [14] to note the ways in which institutional processes of complaint management might be structured to protect the organisation or positively respond to some health consumers and not others. Complaints processes may be more accessible to consumers with greater resources by, for example, being complex and lengthy processes and therefore require more energy and time to achieve resolution. Additionally, people who are stigmatised may feel they have more to lose or may put their fragile access to care at risk by making a complaint at all.

Other constructs of the CFIR “outer setting” which were not apparent in the mechanisms identified by participants included *Local Conditions* and *Local Attitudes*. These issues may be relevant, for example, if there are different levels of support among healthcare executives for implementation of structural responses for stigma reduction. The construct of *Financing* was referred to in relation to the resources needed for genuine engagement with accreditation processes. Issues of *Market Pressure* such as promoting a service as inclusion positive or stigma-free were not mentioned as a desirable marketing element of healthcare organisations. Given the range of experience of this group of participants, they would likely be aware of healthcare organisations which directly or indirectly exclude clients affected by BBV/STIs and how this may impact public perception and service access. In other research, stakeholders within the BBV/STI sector have noted ways in which health services make clear who is not legitimate or welcome by, for example, showing signage in waiting rooms that list medications which will not be prescribed [15].

This paper was aimed at illuminating the possibilities for connecting, or creating strategic collusion between, existing governance processes for quality care and stigma reduction efforts. We focused on participants within the BBV/STI sector, as stigma has been a defining characteristic of the response, and has been noted as a priority since the first Australian HIV strategy in 1989 [46]. We are aware of much stigma focused work occurring in other health sectors and what has been canvassed here might not exhaust the possibilities for collusion within the BBV/STI sector or beyond or might not cover additional concerns or critical views of such collusion. Additionally, while some participants would have also been consumers of health services, there is a need for future research that asks health consumers what they want to see in structural mechanisms for stigma reduction as part of quality care. The analysis presented here cannot substitute for the perspectives of people with direct experience of stigma in healthcare. The CFIR framework was useful for guiding analysis and interpretation in relation to the “outer setting” of healthcare, and for exploring which constructs of this setting are available in participant narratives, and which are absent. The CFIR authors call for continuing development of this framework [32], so other elements that are useful (or critically absent) may appear in future revisions that would extend the analysis presented here.

Typical efforts to address stigma in healthcare (education, information, or contact interventions) do not deliver sustained change, in part due to insufficient resourcing, limited ability to repeat interventions over time and the failure to embed these programs at a structural level. Innovative approaches using other tools, including macro, structural tools provide a promising opportunity to trial. This analysis shows that there may be potential to develop sustained and integrated approaches to stigma reduction in healthcare by aligning with existing governance processes for quality care. Accrediting processes for healthcare organisations and health workers are a fertile area for further investigation including exploration of the conditions which contribute to accreditation being seen or experienced as an effective influence on healthcare organisations.Local processes of governance (performance agreements and the like) and developments in industrial legislation regarding the responsibilities of employers for safe workplaces are other areas to explore. How stigma should be written into these processes, the challenges of implementing structural approaches for real effect and what consumers want from them are areas still to be examined.
