# Supplementary material for: Stigma reduction as quality health care: A qualitative study of structural level mechanisms to address stigma
Source: PLoS One. 2026 Sep 8;21(9):e0357633. doi: 10.1371/journal.pone.0357633 (PMC13552769; doi:10.1371/journal.pone.0357633)
Supplement: Supplementary file 2 [file pone.0357633.s002.docx]

Reviewer #2:

**Comment:**

**The revised version is improved, particularly in the introduction and methods, and the authors have responded constructively to reviewer comments. The use of CFIR “outer setting” constructs provides a clear organizing framework for the analysis, and the findings are presented in a way that is meaningful for policy and practice. The discussion is also appropriately reflective about the promise and limitations of structural approaches.**

**I remain broadly positive about the manuscript, and I think it is close to publishable form. My remaining comments are relatively minor:**

Response:
We thank the reviewer for their comments and support of our paper.

**Comment**:

**Analytic clarity could still be strengthened slightly. While the authors have improved the methods section, the paper would benefit from one or two more sentences clarifying how structural stigma was identified in the data and how inductive insights were linked to the CFIR framework.**

Response

We have added additional text to clarify how we identify structural stigma in the data.

Our understanding of structural stigma in the BBV/STI sector is informed by our previous research speaking to governance processes and accreditation (12) as well as frameworks for understanding and responding to structural stigma in mental health (11).

The following paragraph in the methods section details how inductive insights were linked to the CFIR framework. Accreditation to quality standards is used as one example.

Analysis was guided by the Consolidated Framework for Implementation Research (CFIR) (32) which is a highly cited and widely used conceptual tool for explaining barriers and facilitators to implementation effectiveness (32). The use of CFIR prior to implementation of a program is uncommon (33) but useful to identify factors that can inform development and roll-out of future interventions (34). While activities or mechanisms to support stigma reduction might operate within a healthcare organisation (the “inner setting” as considered by CFIR), we looked for mechanisms that linked to or were determined governing processes (“outer setting”), such as those emerging from the state more broadly. Accreditation to quality standards is one example of a mechanism in the “outer setting” that has been highlighted in the literature on responses to structural stigma (11). We used constructs of the CFIR “outer setting” to categorise other activities and their governance processes that were identified or alluded to by participants (see Table 1 for definitions). In inductive analysis, we examined how participants perceived the utility and application of these mechanisms to support stigma reduction interventions, particularly identifying where participants were critical or sceptical of these aspects of the “outer setting”.

**Comment:**

**The absence of lived-experience perspectives remains an important limitation. This is acknowledged, which is appreciated, but I encourage the authors to state even more clearly that stakeholder perspectives on structural reform cannot substitute for the perspectives of people directly experiencing stigma in healthcare.**

Response:

We agree and have added this to the discussion/limitations paragraph:

The analysis presented here cannot substitute for the perspectives of people with direct experience of stigma in healthcare.

**Comment:**
**There is a small risk of overstatement in some parts of the discussion. The argument is persuasive, but the findings are based on a relatively focused Australian BBV/STI stakeholder sample. Some phrasing could be softened slightly to emphasize that the paper identifies promising structural pathways rather than demonstrating their effectiveness.**

Response:
We agree that there are additional places that we could add some qualifiers and have done so eg (new wording underlined)

In this paper, we examined the perceptions of experienced health workers in relation to possibilities that structural-level governing mechanisms that could be “put to use” and…

Participant discussions of accreditation focused on its potential usefulness to focus the attention of health executives to the matter as an *External Pressure*.

This is a promising step forward and might be useful to direct providers’ attention

This analysis shows that there may be potential to develop sustained and integrated approaches

We point also to existing wording that aimed to present a careful interpretation eg:

Participants’ accounts also raise the possibility of using other more general industrial relations laws

This paper was aimed at illuminating the possibilities for connecting, or creating strategic collusion between

Accrediting processes for healthcare organisations and health workers are a fertile area for further investigation along

**Comment**:
**The discussion of implementation challenges could be sharpened. The manuscript notes the possibility that accreditation and governance processes may become “tick-box” exercises. This is an important point and deserves just a little more emphasis, given that it is central to the paper’s argument.**

Response:

We agree.

We have added this sentence to the first paragraph of the discussion:

Participants also note that while there may be possibility of using these structural supports, there is enduring challenge for genuine implementation beyond “tick and flick” pro forma responses.

And added this detail (in underline) to the conclusion paragraph:

How stigma should be written into these processes, the challenges of implementing structural approaches for real effect and what consumers want from them are areas still to be examined.

**Comment:
Overall, this is a strong and worthwhile manuscript. It offers a valuable conceptual and policy contribution and, in my view, would be suitable for publication after minor final refinements to clarity, caution in claims, and emphasis on the study’s limitations.**

Response:

Thank you again for helping us strengthen this paper.
