# Supplementary material for: Stigma reduction as quality health care: A qualitative study of structural level mechanisms to address stigma
Source: PLoS One. 2026 Sep 8;21(9):e0357633. doi: 10.1371/journal.pone.0357633 (PMC13552769; doi:10.1371/journal.pone.0357633)
Supplement: Supplementary file 3 [file pone.0357633.s001.docx]

**Journal Requirements:**

When submitting your revision, we need you to address these additional requirements.

**1. Please ensure that your manuscript meets PLOS ONE's style requirements, including those for file naming. The PLOS ONE style templates can be found at** <https://journals.plos.org/plosone/s/file?id=wjVg/PLOSOne_formatting_sample_main_body.pdf> and <https://journals.plos.org/plosone/s/file?id=ba62/PLOSOne_formatting_sample_title_authors_affiliations.pdf>

We have checked the style requirements and made relevant changes.

**2. We note that you have indicated that there are restrictions to data sharing for this study. For studies involving human research participant data or other sensitive data, we encourage authors to share de-identified or anonymized data. However, when data cannot be publicly shared for ethical reasons, we allow authors to make their data sets available upon request. For information on unacceptable data access restrictions, please see** [**http://journals.plos.org/plosone/s/data-availability#loc-unacceptable-data-access-restrictions**](http://journals.plos.org/plosone/s/data-availability#loc-unacceptable-data-access-restrictions)**.

Before we proceed with your manuscript, please address the following prompts:

a) If there are ethical or legal restrictions on sharing a de-identified data set, please explain them in detail (e.g., data contain potentially identifying or sensitive patient information, data are owned by a third-party organization, etc.) and who has imposed them (e.g., a Research Ethics Committee or Institutional Review Board, etc.). Please also provide contact information for a data access committee, ethics committee, or other institutional body to which data requests may be sent.

b) If there are no restrictions, please upload the minimal anonymized data set necessary to replicate your study findings to a stable, public repository and provide us with the relevant URLs, DOIs, or accession numbers. Please see** [**http://www.bmj.com/content/340/bmj.c181.long**](http://www.bmj.com/content/340/bmj.c181.long) **for guidelines on how to de-identify and prepare clinical data for publication. For a list of recommended repositories, please see** [**https://journals.plos.org/plosone/s/recommended-repositories**](https://journals.plos.org/plosone/s/recommended-repositories)**. You also have the option of uploading the data as Supporting Information files, but we would recommend depositing data directly to a data repository if possible.

Please update your Data Availability statement in the submission form accordingly.**

We have amended the information provided to include details of UNSW Human Research Ethics Committee:

Data are not available to be shared. Participants recount information about their own

experiences of stigma in a way that cannot be adequately deidentified. Ethics

Committee approval did not include sharing of data. UNSW Sydney Human Research Ethics Committee can be contacted on  humanethics@unsw.edu.au or by phone at +61 2 9385 6222, citing project number: HC220669.

**3. Please provide a complete Data Availability Statement in the submission form, ensuring you include all necessary access information or a reason for why you are unable to make your data freely accessible. If your research concerns only data provided within your submission, please write "All data are in the manuscript and/or supporting information files" as your Data Availability Statement.**

We have added detail as above.

**4. Thank you for stating the following in the Competing Interests section:
I have read the journal's policy and the authors of this manuscript have the following competing interests:**

**CT has received speakers honorarium from Gilead Sciences. MS has received investigator-initiated research funding from Gilead Sciences and AbbVie and consultant fees from Gilead Sciences.

Please confirm that this does not alter your adherence to all PLOS ONE policies on sharing data and materials, by including the following statement: "This does not alter our adherence to PLOS ONE policies on sharing data and materials.” (as detailed online in our guide for authors** [**http://journals.plos.org/plosone/s/competing-interests**](http://journals.plos.org/plosone/s/competing-interests)**). If there are restrictions on sharing of data and/or materials, please state these. Please note that we cannot proceed with consideration of your article until this information has been declared.

Please include your updated Competing Interests statement in your cover letter; we will change the online submission form on your behalf.**

We have added the relevant information to the cover letter as requested.

**5. We note that the grant information you provided in the ‘Funding Information’ and ‘Financial Disclosure’ sections do not match.

When you resubmit, please ensure that you provide the correct grant numbers for the awards you received for your study in the ‘Funding Information’ section.**

We have provided financial information as requested.

**6. If the reviewer comments include a recommendation to cite specific previously published works, please review and evaluate these publications to determine whether they are relevant and should be cited. There is no requirement to cite these works unless the editor has indicated otherwise.**

There were no such recommendations for reviewers.

**7. Please review your reference list to ensure that it is complete and correct. If you have cited papers that have been retracted, please include the rationale for doing so in the manuscript text, or remove these references and replace them with relevant current references. Any changes to the reference list should be mentioned in the rebuttal letter that accompanies your revised manuscript. If you need to cite a retracted article, indicate the article’s retracted status in the References list and also include a citation and full reference for the retraction notice.**

We have reviewed the reference list to ensure that it is complete and correct.

Reviewers' comments:

Reviewer's Responses to Questions

**Comments to the Author**

1. Is the manuscript technically sound, and do the data support the conclusions?

The manuscript must describe a technically sound piece of scientific research with data that supports the conclusions. Experiments must have been conducted rigorously, with appropriate controls, replication, and sample sizes. The conclusions must be drawn appropriately based on the data presented.

Reviewer #1: Yes

Reviewer #2: Yes

Reviewer #3: Partly

2. Has the statistical analysis been performed appropriately and rigorously?

Reviewer #1: N/A

Reviewer #2: N/A

Reviewer #3: N/A

3. Have the authors made all data underlying the findings in their manuscript fully available?

The [PLOS Data policy](http://track.editorialmanager.com/CL0/http:%2F%2Fwww.plosone.org%2Fstatic%2Fpolicies.action%23sharing/1/010f019bedf05c08-5c94d6ef-86a2-498a-8fad-5205ce6d9b01-000000/apCgU6w4RwhY0sYEkR8ClEtbN_zs0eG5mceK_acMMz8=247) requires authors to make all data underlying the findings described in their manuscript fully available without restriction, with rare exception (please refer to the Data Availability Statement in the manuscript PDF file). The data should be provided as part of the manuscript or its supporting information, or deposited to a public repository. For example, in addition to summary statistics, the data points behind means, medians and variance measures should be available. If there are restrictions on publicly sharing data—e.g. participant privacy or use of data from a third party—those must be specified.

Reviewer #1: Yes

Reviewer #2: Yes

Reviewer #3: No

4. Is the manuscript presented in an intelligible fashion and written in standard English?

PLOS ONE does not copyedit accepted manuscripts, so the language in submitted articles must be clear, correct, and unambiguous. Any typographical or grammatical errors should be corrected at revision, so please note any specific errors here.

Reviewer #1: Yes

Reviewer #2: Yes

Reviewer #3: Yes

5. Review Comments to the Author

Please use the space provided to explain your answers to the questions above. You may also include additional comments for the author, including concerns about dual publication, research ethics, or publication ethics. (Please upload your review as an attachment if it exceeds 20,000 characters)

**Reviewer #1: Dear Authors,
I commend the your efforts to investigate the structural-level mechanisms for stigma reduction utilizing a qualitative methodology. Although the study did not include consumers as participants, the insights gleaned from other key stakeholders significantly contribute to the understanding of structural stigma reduction. The following comments are offered to facilitate the manuscript's improvisation:**

Thank you for positive comments and suggestions for improving our manuscript.

**General and Language-related comments
The coherence and flow of the paper require strengthening. Specifically, the connection between paragraphs, particularly within the introduction, should be made more explicit to ensure a smoother narrative progression.**
Thank you for this suggestion. We have made numerous edits through the introduction to connect paragraphs.

**Introduction Section**
**• It is imperative to define stigma in greater detail from a structural and governance perspective within the introduction. Furthermore, the authors must clearly articulate how stigma is operationalized for the current study.**
Thank you for this helpful comment. We have substantially re-written and re-organised the introduction to clarify our approach to issues of structural stigma and governance mechanisms.

**• A typographical error appears to be present in line 81 on page 5 of the manuscript and requires correction.**
This sentence has now been deleted.

Methods Section
**• While acknowledging this study is part of a larger stigma reduction project, the authors are advised to explicitly specify the qualitative design or approach employed for this specific study within the Methods section.**

We have specified the qualitative design in the Methods section.

**• The use of adjectives such as 'high-level' insights (mentioned in line 167) should be avoided unless it is substantiated by relevant literature.**

This term has been removed from the manuscript.

**• It would be beneficial to state whether the authors utilized a-priori inclusion criteria for the selection of participants.**
We have added in further detail on the types of characteristics we were looking for in purposive sampling of participants. New detail is underlined.

The team sought to ensure a spread of expertise in health and community settings, representing Australian jurisdictions, a balance of experience and knowledge across viral hepatitis, HIV and other STIs, and experience working in urban, outer urban and regional contexts. The stakeholders held current or previous roles in policy, management and service provision across primary, community and tertiary settings, in peer-led and identified roles and organisations, professional associations and research.

**• I recommend providing a selection of broad questions from the semi-structured interview guide used during the data collection process.**
We have added in further information about the topics in the interview guide. New details are underlined:

In alignment with the aims of the project, stakeholders were asked to reflect on and respond to the concept of a ‘universal precautions’ approach to stigma reduction. This included topics such as the acceptability of the term, the feasibility of developing, designing and implementing a universal framework to stigma reduction and measuring change. They were asked to identify existing structures, policies, procedures, and frameworks where there is capacity to embed stigma reduction interventions.

**• The pragmatic approach adopted for data analysis is considered well-justified.**
Thank you

Results and Discussion Section
**• The Results section lacks a description pertaining to financing and societal pressures. The authors should reflect on these constructs in the discussion. If data related to these constructs did not emerge during the interviews, it is important to reflect on it.**
We note a typo on “societal pressure” (which was incorrectly labelled as “social pressures). There are two paragraphs in the discussion reflecting on CFIR constructs which were not apparent in the data (notably societal pressure and market pressure). We have added in comment on other Outer Setting constructs which were not apparent in the data analysed here.

**• Considering the migration rate in Australia, integrating stigma reduction strategies at the structural level aimed at improving healthcare quality for Culturally and Linguistically Diverse (CALD) communities within the discussion section would substantially enhance the manuscript’s value and relevance.**

While we agree with the reviewer regarding the need for better quality (and less stigmatising care) for CALD communities, we are approaching this project with the goal of improving care for all people. It could be argued that many groups (including First Nation peoples) could benefit from stigma free care, so we have chosen not to highlight any one particular group or identity.

**Reviewer #2: This qualitative study evaluates how stigma reduction can be embedded within existing healthcare quality and governance structures. Based on interviews with 20 key stakeholders in the Australian BBV/STI sector and guided by the CFIR “outer setting”, the manuscript identifies accreditation, quality standards, performance pressures, and workplace legislation as structural levers for sustainable stigma reduction. The paper argues that aligning stigma reduction with quality care mechanisms suggests a more effective alternative to individual-level interventions.
This manuscript provides a timely and persuasive framing of stigma as a quality-of-care issue and makes a strong case for focusing on structural rather than purely individual-level responses. The use of CFIR, particularly the emphasis on “outer setting” mechanisms, is appropriate and clearly supports the analysis. The study draws on rich qualitative data from highly experienced stakeholders, and the findings are clearly relevant to policy and practice, especially in relation to accreditation and governance processes within healthcare systems.**

Thank you for these positive comments.

**However, the absence of lived-experience perspectives limits the breadth of insight, and generalisation to other areas of healthcare should be made cautiously.**

The limitations section included comment that additional work is needed with consumers of health services to further explore perspectives of lived/living experience.

**In addition, some recommendations remain at a relatively high policy level, with less attention to how they might be translated into everyday practice or how risks of superficial, “tick-box” implementation could be addressed.**

We agree. But these translations into everyday practice are beyond the scope of this paper. We included a specific focus on “critical perspectives” in the analysis method to interrogate the potential limitations of a structural approach.

**The manuscript would also benefit from light editing to simplify some of the longer and denser sentences to improve readability.**

Thank you – we have undertaken a thorough edit.

**Reviewer #3: This manuscript, exploring structural level mechanisms to address stigma, is about an important subject from an interesting perspective. This qualitative study has several strengths, but the manuscript also shows some weaknesses that I will try to point out below. I believe that a careful rewrite of some parts of the manuscript will improve its quality.**

My comments:
**1. The introduction, methods and discussion sessions contain a lot of, but important, text. For me, it was sometimes a little difficult to navigate the text. Consider adding subheadings to make it easier for the reader.**

Thank you for the prompt. We have reduced the complexity and the length of the introduction to improve readability.

**2. Please, review the language throughout the manuscript. During my reading, I found sentences that were incomplete (eg. Page 5, line 84 and page 11, lines 211-212), very long (e.g. page 14, lines 299-305) and xxx (e.g. xxx).**

Thank you for those examples. We have reviewed and edited the paper for readability.

**3. The methods section needs more information about how the study was conducted. Consider using COREQ or other guideline for qualitative research to guide the presentation of the method.
a. The text about recruitment (page 8) needs more information, e.g. how many were invited, how many declined participation and for what reason?**

We have added in the following information:

All stakeholders agreed to participate, however due to travel and international time differences, one interview did not transpire, and a suitable representative from the service was interviewed.

**b. The description about the data collection also needs to be elaborated. E.g. where and how were the interviews conducted (face-to-face/telephone/digital)? How long were the interviews? Please include the interview schedule, or at least the main questions.**
We have added in the following information regarding how interviews were conducted.

All interviews were conducted via Zoom or Teams with the video function turned on and were between 50 – 100 minutes in length.

See above regarding interview schedule.

**c. The analysis is poorly described, which makes it difficult to understand the result. E.g. the different steps of the analysis are unclear and need to be described, it is also unclear what steps were inductive vs deductive.**
We have clarified the description of the analysis method.

**d. I think I can understand the thought behind using a framework related to implementation science, but this needs to be clearly motivated in the manuscript.**
We have strengthened reference to the possibilities of implementing stigma reduction at structural levels to connect the logic throughout the paper.

**4. As I wrote above, it was difficult to follow the result text when the analysis process was unclear. All constructs from the “outer setting” in CFIR are not represented in the result text. Does that mean that no data in the interview was related to those constructs? A table with an overview of the analysis process and/or an overview of the final analysis would be helpful for the reader.**

We have added reference to all constructs in the discussion to indicate those which did not feature in participant accounts.

**5. The part on page 11, lines 229-235 – it is unclear if it is the participants view or if it is information about the setting (that then should be moved to the methods section).**
Thank you – we have clarified that this is drawn from the data.

**6. A small detail - observe that on page 16, lines 354-355 you have written “to be implemented mid-2025”. Since it is now 2026, please update the text.**

This is updated.
