# Supplementary material for: Stigma reduction as quality health care: A qualitative study of structural level mechanisms to address stigma
Source: PLoS One. 2026 Sep 8;21(9):e0357633. doi: 10.1371/journal.pone.0357633 (PMC13552769; doi:10.1371/journal.pone.0357633)
Supplement: Supplementary file 4 [file pone.0357633.s003.docx]

**Comment:**

**Reviewer #2: This is a strong and policy-relevant qualitative manuscript. The study makes a valuable contribution by reframing stigma reduction as part of quality health care and by identifying structural mechanisms, particularly accreditation and governance processes, that may be mobilized to support stigma reduction. The use of CFIR outer-setting constructs provides a coherent analytic framework, and the findings are clearly linked to implications for health-system practice.**

**The revisions have adequately addressed most major concerns. The methods are now clearer, the authors have acknowledged the absence of lived-experience perspectives, and the discussion has appropriately softened causal or overly strong claims. I have only minor remaining suggestions.**

Response:

Thank for you for the support of our paper

**Comment:**

**First, the manuscript should continue to emphasize that the findings identify promising structural pathways rather than demonstrating their effectiveness.**

Response:

We note existing cautions language:

- Abstract: Innovative approaches to stigma reduction using macro, structural tools provide a promising opportunity to trial.
- Methods: more needs to be known about the feasibility of these structural mechanisms for addressing the stigmatisation of BBV/STIs and beyond.
- Results: Accreditation requirements (*Policies and Laws* within the “outer setting”) were seen to have the potential to drive the management team
- Results: Participants noted that regular processes of institutional management and workforce development could be adapted
- Discussion: examined the perceptions of experienced health workers in relation to possibilities that structural-level governing mechanisms
- Discussion: also raise the possibility of using other more general industrial relations laws
- Discussion: This paper was aimed at illuminating the possibilities for

We have added new wording emphasizing the possibility of these pathways:

- From line 215 - *Policies and Laws* as well as *Performance Measurement Pressure* were reflected in participant comments about the possibility of driving change by embedding stigma
- From line 322 - Within the workplace, *Performance Measurement Pressure* (CFIR “outer setting” construct) for individual workers was also recognised as a possible means to support stigma reduction
- From line 345 - Participant accounts support the argument for developing and testing a strategic collusion, a systematic and deliberate bringing together,

**Comment:**

**Second, the limitation regarding the absence of direct patient/consumer perspectives should remain explicit.**

Response:

We note that this limitation is already included and explicit (from line 420, in limitations section):

Additionally, while some participants would have also been consumers of health services, there is a need for future research that asks health consumers what they want to see in structural mechanisms for stigma reduction as part of quality care. The analysis presented here cannot substitute for the perspectives of people with direct experience of stigma in healthcare.

And from line 440, in concluding paragraph:

How stigma should be written into these processes, the challenges of implementing structural approaches for real effect and what consumers want from them are areas still to be examined.

**Comment:**

**Third, the discussion of implementation challenges, especially the risk that accreditation becomes a “tick-box” exercise, could be sharpened slightly.**

Response:

We have added emphasis to the concluding paragraph:

- Accrediting processes for healthcare organisations and health workers are a fertile area for further investigation including exploration of the conditions which contribute to accreditation being seen or experienced as an effective influence on healthcare organisations.

**Finally, claims about transferability beyond the Australian BBV/STI context should be phrased cautiously.**

Response:

There is only one mention of transferability of this analysis beyond the current context. We note existing cautious language in this section:

- In this way, features of the health system that were identified as useful for BBVs/STIs may be applicable and adaptable for other conditions, identities, and practices.

We have strengthened the language:

- Features of the health system that were identified as useful for BBVs/STIs could be explored for their applicability and adaptability for other conditions, identities, and practices.
