# Supplementary material for: Stigma reduction as quality health care: A qualitative study of structural level mechanisms to address stigma
Source: PLoS One. 2026 Sep 8;21(9):e0357633. doi: 10.1371/journal.pone.0357633 (PMC13552769; doi:10.1371/journal.pone.0357633)
Supplement: Peer review history

# Peer review: Decision Letter 0

**DOI:** 10.1371/journal.pone.0357633.r001

**Authors:** Gamji Rabiu Abu-Ba'are

23 Jan 2026

Dear Dr. Treloar,

plosone@plos.org. When you're ready to submit your revision, log on to https://www.editorialmanager.com/pone/ and select the 'Submissions Needing Revision' folder to locate your manuscript file.

- A letter that responds to each point raised by the academic editor and reviewer(s). You should upload this letter as a separate file labeled 'Response to Reviewers'.
- A marked-up copy of your manuscript that highlights changes made to the original version. You should upload this as a separate file labeled 'Revised Manuscript with Track Changes'.
- An unmarked version of your revised paper without tracked changes. You should upload this as a separate file labeled 'Manuscript'.

If applicable, we recommend that you deposit your laboratory protocols in protocols.io to enhance the reproducibility of your results. Protocols.io assigns your protocol its own identifier (DOI) so that it can be cited independently in the future. For instructions see: https://journals.plos.org/plosone/s/submission-guidelines#loc-laboratory-protocols. Additionally, PLOS ONE offers an option for publishing peer-reviewed Lab Protocol articles, which describe protocols hosted on protocols.io. Read more information on sharing protocols at https://plos.org/protocols?utm_medium=editorial-email&utm_source=authorletters&utm_campaign=protocols.

We look forward to receiving your revised manuscript.

Kind regards,

Gamji Rabiu Abu-Ba'are, Ph.D, MA

Academic Editor

PLOS One

Journal Requirements:

When submitting your revision, we need you to address these additional requirements.

1. Please ensure that your manuscript meets PLOS ONE's style requirements, including those for file naming. The PLOS ONE style templates can be found at https://journals.plos.org/plosone/s/file?id=wjVg/PLOSOne_formatting_sample_main_body.pdf and https://journals.plos.org/plosone/s/file?id=ba62/PLOSOne_formatting_sample_title_authors_affiliations.pdf

2. We note that you have indicated that there are restrictions to data sharing for this study. For studies involving human research participant data or other sensitive data, we encourage authors to share de-identified or anonymized data. However, when data cannot be publicly shared for ethical reasons, we allow authors to make their data sets available upon request. For information on unacceptable data access restrictions, please see http://journals.plos.org/plosone/s/data-availability#loc-unacceptable-data-access-restrictions.

Before we proceed with your manuscript, please address the following prompts:

a) If there are ethical or legal restrictions on sharing a de-identified data set, please explain them in detail (e.g., data contain potentially identifying or sensitive patient information, data are owned by a third-party organization, etc.) and who has imposed them (e.g., a Research Ethics Committee or Institutional Review Board, etc.). Please also provide contact information for a data access committee, ethics committee, or other institutional body to which data requests may be sent.

b) If there are no restrictions, please upload the minimal anonymized data set necessary to replicate your study findings to a stable, public repository and provide us with the relevant URLs, DOIs, or accession numbers. Please see http://www.bmj.com/content/340/bmj.c181.long for guidelines on how to de-identify and prepare clinical data for publication. For a list of recommended repositories, please see https://journals.plos.org/plosone/s/recommended-repositories. You also have the option of uploading the data as Supporting Information files, but we would recommend depositing data directly to a data repository if possible.

Please update your Data Availability statement in the submission form accordingly.

3. Please provide a complete Data Availability Statement in the submission form, ensuring you include all necessary access information or a reason for why you are unable to make your data freely accessible. If your research concerns only data provided within your submission, please write "All data are in the manuscript and/or supporting information files" as your Data Availability Statement.

4. Thank you for stating the following in the Competing Interests section:

I have read the journal's policy and the authors of this manuscript have the following competing interests:

CT has received speakers honorarium from Gilead Sciences. MS has received investigator-initiated research funding from Gilead Sciences and AbbVie and consultant fees from Gilead Sciences.

Please confirm that this does not alter your adherence to all PLOS ONE policies on sharing data and materials, by including the following statement: "This does not alter our adherence to PLOS ONE policies on sharing data and materials.” (as detailed online in our guide for authors http://journals.plos.org/plosone/s/competing-interests). If there are restrictions on sharing of data and/or materials, please state these. Please note that we cannot proceed with consideration of your article until this information has been declared.

Please include your updated Competing Interests statement in your cover letter; we will change the online submission form on your behalf.

5. We note that the grant information you provided in the ‘Funding Information’ and ‘Financial Disclosure’ sections do not match.

When you resubmit, please ensure that you provide the correct grant numbers for the awards you received for your study in the ‘Funding Information’ section.

6. If the reviewer comments include a recommendation to cite specific previously published works, please review and evaluate these publications to determine whether they are relevant and should be cited. There is no requirement to cite these works unless the editor has indicated otherwise.

7. Please review your reference list to ensure that it is complete and correct. If you have cited papers that have been retracted, please include the rationale for doing so in the manuscript text, or remove these references and replace them with relevant current references. Any changes to the reference list should be mentioned in the rebuttal letter that accompanies your revised manuscript. If you need to cite a retracted article, indicate the article’s retracted status in the References list and also include a citation and full reference for the retraction notice.

Additional Editor Comments :

Dear Author, Thank you for submitting you important work for consideration with PLOS ONE. Overall reviewers had favorable comments and found your paper to have merits, however, there are some minor comments they recommend you address. Please respond to the review comments and return. Congratulations.

[Note: HTML markup is below. Please do not edit.]

Reviewers' comments:

Reviewer's Responses to Questions

**Comments to the Author**

1. Is the manuscript technically sound, and do the data support the conclusions?

Reviewer #1: Yes

Reviewer #2: Yes

Reviewer #3: Partly

**********

2. Has the statistical analysis been performed appropriately and rigorously?

Reviewer #1: N/A

Reviewer #2: N/A

Reviewer #3: N/A

**********

3. Have the authors made all data underlying the findings in their manuscript fully available?

The PLOS Data policy

Reviewer #1: Yes

Reviewer #2: Yes

Reviewer #3: No

**********

4. Is the manuscript presented in an intelligible fashion and written in standard English?

Reviewer #1: Yes

Reviewer #2: Yes

Reviewer #3: Yes

**********

Reviewer #1: Dear Authors,

I commend the your efforts to investigate the structural-level mechanisms for stigma reduction utilizing a qualitative methodology. Although the study did not include consumers as participants, the insights gleaned from other key stakeholders significantly contribute to the understanding of structural stigma reduction. The following comments are offered to facilitate the manuscript's improvisation:

General and Language-related comments

The coherence and flow of the paper require strengthening. Specifically, the connection between paragraphs, particularly within the introduction, should be made more explicit to ensure a smoother narrative progression.

Introduction Section

• It is imperative to define stigma in greater detail from a structural and governance perspective within the introduction. Furthermore, the authors must clearly articulate how stigma is operationalized for the current study.

• A typographical error appears to be present in line 81 on page 5 of the manuscript and requires correction.

Methods Section

• While acknowledging this study is part of a larger stigma reduction project, the authors are advised to explicitly specify the qualitative design or approach employed for this specific study within the Methods section.

• The use of adjectives such as 'high-level' insights (mentioned in line 167) should be avoided unless it is substantiated by relevant literature.

• It would be beneficial to state whether the authors utilized a-priori inclusion criteria for the selection of participants.

• I recommend providing a selection of broad questions from the semi-structured interview guide used during the data collection process.

• The pragmatic approach adopted for data analysis is considered well-justified.

Results and Discussion Section

• The Results section lacks a description pertaining to financing and societal pressures. The authors should reflect on these constructs in the discussion. If data related to these constructs did not emerge during the interviews, it is important to reflect on it.

• Considering the migration rate in Australia, integrating stigma reduction strategies at the structural level aimed at improving healthcare quality for Culturally and Linguistically Diverse (CALD) communities within the discussion section would substantially enhance the manuscript's value and relevance.

Reviewer #2: This qualitative study evaluates how stigma reduction can be embedded within existing healthcare quality and governance structures. Based on interviews with 20 key stakeholders in the Australian BBV/STI sector and guided by the CFIR “outer setting”, the manuscript identifies accreditation, quality standards, performance pressures, and workplace legislation as structural levers for sustainable stigma reduction. The paper argues that aligning stigma reduction with quality care mechanisms suggests a more effective alternative to individual-level interventions.

This manuscript provides a timely and persuasive framing of stigma as a quality-of-care issue and makes a strong case for focusing on structural rather than purely individual-level responses. The use of CFIR, particularly the emphasis on “outer setting” mechanisms, is appropriate and clearly supports the analysis. The study draws on rich qualitative data from highly experienced stakeholders, and the findings are clearly relevant to policy and practice, especially in relation to accreditation and governance processes within healthcare systems.

However, the absence of lived-experience perspectives limits the breadth of insight, and generalisation to other areas of healthcare should be made cautiously. In addition, some recommendations remain at a relatively high policy level, with less attention to how they might be translated into everyday practice or how risks of superficial, “tick-box” implementation could be addressed. The manuscript would also benefit from light editing to simplify some of the longer and denser sentences to improve readability.

Reviewer #3: This manuscript, exploring structural level mechanisms to address stigma, is about an important subject from an interesting perspective. This qualitative study has several strengths, but the manuscript also shows some weaknesses that I will try to point out below. I believe that a careful rewrite of some parts of the manuscript will improve its quality.

My comments:

1. The introduction, methods and discussion sessions contain a lot of, but important, text. For me, it was sometimes a little difficult to navigate the text. Consider adding subheadings to make it easier for the reader.

2. Please, review the language throughout the manuscript. During my reading, I found sentences that were incomplete (eg. Page 5, line 84 and page 11, lines 211-212), very long (e.g. page 14, lines 299-305) and xxx (e.g. xxx).

3. The methods section needs more information about how the study was conducted. Consider using COREQ or other guideline for qualitative research to guide the presentation of the method.

a. The text about recruitment (page 8) needs more information, e.g. how many were invited, how many declined participation and for what reason?

b. The description about the data collection also needs to be elaborated. E.g. where and how were the interviews conducted (face-to-face/telephone/digital)? How long were the interviews? Please include the interview schedule, or at least the main questions.

c. The analysis is poorly described, which makes it difficult to understand the result. E.g. the different steps of the analysis are unclear and need to be described, it is also unclear what steps were inductive vs deductive.

d. I think I can understand the thought behind using a framework related to implementation science, but this needs to be clearly motivated in the manuscript.

4. As I wrote above, it was difficult to follow the result text when the analysis process was unclear. All constructs from the “outer setting” in CFIR are not represented in the result text. Does that mean that no data in the interview was related to those constructs? A table with an overview of the analysis process and/or an overview of the final analysis would be helpful for the reader.

5. The part on page 11, lines 229-235 – it is unclear if it is the participants view or if it is information about the setting (that then should be moved to the methods section).

6. A small detail - observe that on page 16, lines 354-355 you have written “to be implemented mid-2025”. Since it is now 2026, please update the text.

**********

what does this mean?). If published, this will include your full peer review and any attached files.

If you choose “no”, your identity will remain anonymous but your review may still be made public.

**Do you want your identity to be public for this peer review?**  For information about this choice, including consent withdrawal, please see our Privacy Policy

Reviewer #1: No

Reviewer #2: No

Reviewer #3: No

**********

[NOTE: If reviewer comments were submitted as an attachment file, they will be attached to this email and accessible via the submission site. Please log into your account, locate the manuscript record, and check for the action link "View Attachments". If this link does not appear, there are no attachment files.]

To ensure your figures meet our technical requirements, please review our figure guidelines: https://journals.plos.org/plosone/s/figures

You may also use PLOS’s free figure tool, NAAS, to help you prepare publication quality figures: https://journals.plos.org/plosone/s/figures#loc-tools-for-figure-preparation.

NAAS will assess whether your figures meet our technical requirements by comparing each figure against our figure specifications.

# Author comment: Author response to Decision Letter 1

**DOI:** 10.1371/journal.pone.0357633.r002

6 Mar 2026

Journal Requirements:

When submitting your revision, we need you to address these additional requirements.

1. Please ensure that your manuscript meets PLOS ONE's style requirements, including those for file naming. The PLOS ONE style templates can be found at https://journals.plos.org/plosone/s/file?id=wjVg/PLOSOne_formatting_sample_main_body.pdf and https://journals.plos.org/plosone/s/file?id=ba62/PLOSOne_formatting_sample_title_authors_affiliations.pdf

We have checked the style requirements and made relevant changes.

2. We note that you have indicated that there are restrictions to data sharing for this study. For studies involving human research participant data or other sensitive data, we encourage authors to share de-identified or anonymized data. However, when data cannot be publicly shared for ethical reasons, we allow authors to make their data sets available upon request. For information on unacceptable data access restrictions, please see http://journals.plos.org/plosone/s/data-availability#loc-unacceptable-data-access-restrictions.

Before we proceed with your manuscript, please address the following prompts:

a) If there are ethical or legal restrictions on sharing a de-identified data set, please explain them in detail (e.g., data contain potentially identifying or sensitive patient information, data are owned by a third-party organization, etc.) and who has imposed them (e.g., a Research Ethics Committee or Institutional Review Board, etc.). Please also provide contact information for a data access committee, ethics committee, or other institutional body to which data requests may be sent.

b) If there are no restrictions, please upload the minimal anonymized data set necessary to replicate your study findings to a stable, public repository and provide us with the relevant URLs, DOIs, or accession numbers. Please see http://www.bmj.com/content/340/bmj.c181.long for guidelines on how to de-identify and prepare clinical data for publication. For a list of recommended repositories, please see https://journals.plos.org/plosone/s/recommended-repositories. You also have the option of uploading the data as Supporting Information files, but we would recommend depositing data directly to a data repository if possible.

Please update your Data Availability statement in the submission form accordingly.

We have amended the information provided to include details of UNSW Human Research Ethics Committee:

Data are not available to be shared. Participants recount information about their own

experiences of stigma in a way that cannot be adequately deidentified. Ethics

Committee approval did not include sharing of data. UNSW Sydney Human Research Ethics Committee can be contacted on humanethics@unsw.edu.au or by phone at +61 2 9385 6222, citing project number: HC220669.

3. Please provide a complete Data Availability Statement in the submission form, ensuring you include all necessary access information or a reason for why you are unable to make your data freely accessible. If your research concerns only data provided within your submission, please write "All data are in the manuscript and/or supporting information files" as your Data Availability Statement.

We have added detail as above.

4. Thank you for stating the following in the Competing Interests section:

I have read the journal's policy and the authors of this manuscript have the following competing interests:

CT has received speakers honorarium from Gilead Sciences. MS has received investigator-initiated research funding from Gilead Sciences and AbbVie and consultant fees from Gilead Sciences.

Please confirm that this does not alter your adherence to all PLOS ONE policies on sharing data and materials, by including the following statement: "This does not alter our adherence to PLOS ONE policies on sharing data and materials.” (as detailed online in our guide for authors http://journals.plos.org/plosone/s/competing-interests). If there are restrictions on sharing of data and/or materials, please state these. Please note that we cannot proceed with consideration of your article until this information has been declared.

Please include your updated Competing Interests statement in your cover letter; we will change the online submission form on your behalf.

We have added the relevant information to the cover letter as requested.

5. We note that the grant information you provided in the ‘Funding Information’ and ‘Financial Disclosure’ sections do not match.

When you resubmit, please ensure that you provide the correct grant numbers for the awards you received for your study in the ‘Funding Information’ section.

We have provided financial information as requested.

6. If the reviewer comments include a recommendation to cite specific previously published works, please review and evaluate these publications to determine whether they are relevant and should be cited. There is no requirement to cite these works unless the editor has indicated otherwise.

There were no such recommendations for reviewers.

7. Please review your reference list to ensure that it is complete and correct. If you have cited papers that have been retracted, please include the rationale for doing so in the manuscript text, or remove these references and replace them with relevant current references. Any changes to the reference list should be mentioned in the rebuttal letter that accompanies your revised manuscript. If you need to cite a retracted article, indicate the article’s retracted status in the References list and also include a citation and full reference for the retraction notice.

We have reviewed the reference list to ensure that it is complete and correct.

Reviewers' comments:

Reviewer's Responses to Questions

Comments to the Author

1. Is the manuscript technically sound, and do the data support the conclusions?

The manuscript must describe a technically sound piece of scientific research with data that supports the conclusions. Experiments must have been conducted rigorously, with appropriate controls, replication, and sample sizes. The conclusions must be drawn appropriately based on the data presented.

Reviewer #1: Yes

Reviewer #2: Yes

Reviewer #3: Partly

________________________________________

2. Has the statistical analysis been performed appropriately and rigorously?

Reviewer #1: N/A

Reviewer #2: N/A

Reviewer #3: N/A

________________________________________

3. Have the authors made all data underlying the findings in their manuscript fully available?

The PLOS Data policy requires authors to make all data underlying the findings described in their manuscript fully available without restriction, with rare exception (please refer to the Data Availability Statement in the manuscript PDF file). The data should be provided as part of the manuscript or its supporting information, or deposited to a public repository. For example, in addition to summary statistics, the data points behind means, medians and variance measures should be available. If there are restrictions on publicly sharing data—e.g. participant privacy or use of data from a third party—those must be specified.

Reviewer #1: Yes

Reviewer #2: Yes

Reviewer #3: No

________________________________________

4. Is the manuscript presented in an intelligible fashion and written in standard English?

PLOS ONE does not copyedit accepted manuscripts, so the language in submitted articles must be clear, correct, and unambiguous. Any typographical or grammatical errors should be corrected at revision, so please note any specific errors here.

Reviewer #1: Yes

Reviewer #2: Yes

Reviewer #3: Yes

________________________________________

5. Review Comments to the Author

Please use the space provided to explain your answers to the questions above. You may also include additional comments for the author, including concerns about dual publication, research ethics, or publication ethics. (Please upload your review as an attachment if it exceeds 20,000 characters)

Reviewer #1: Dear Authors,

I commend the your efforts to investigate the structural-level mechanisms for stigma reduction utilizing a qualitative methodology. Although the study did not include consumers as participants, the insights gleaned from other key stakeholders significantly contribute to the understanding of structural stigma reduction. The following comments are offered to facilitate the manuscript's improvisation:

Thank you for positive comments and suggestions for improving our manuscript.

General and Language-related comments

The coherence and flow of the paper require strengthening. Specifically, the connection between paragraphs, particularly within the introduction, should be made more explicit to ensure a smoother narrative progression.

Thank you for this suggestion. We have made numerous edits through the introduction to connect paragraphs.

Introduction Section

• It is imperative to define stigma in greater detail from a structural and governance perspective within the introduction. Furthermore, the authors must clearly articulate how stigma is operationalized for the current study.

Thank you for this helpful comment. We have substantially re-written and re-organised the introduction to clarify our approach to issues of structural stigma and governance mechanisms.

• A typographical error appears to be present in line 81 on page 5 of the manuscript and requires correction.

This sentence has now been deleted.

Methods Section

• While acknowledging this study is part of a larger stigma reduction project, the authors are advised to explicitly specify the qualitative design or approach employed for this specific study within the Methods section.

We have specified the qualitative design in the Methods section.

• The use of adjectives such as 'high-level' insights (mentioned in line 167) should be avoided unless it is substantiated by relevant literature.

This term has been removed from the manuscript.

• It would be beneficial to state whether the authors utilized a-priori inclusion criteria for the selection of participants.

We have added in further detail on the types of characteristics we were looking for in purposive sampling of participants. New detail is underlined.

The team sought to ensure a spread of expertise in health and community settings, representing Australian jurisdictions, a balance of experience and knowledge across viral hepatitis, HIV and other STIs, and experience working in urban, outer urban and regional contexts. The stakeholders held current or previous roles in policy, management and service provision across primary, community and tertiary settings, in peer-led and identified roles and organisations, professional associations and research.

• I recommend providing a selection of broad questions from the semi-structured interview guide used during the data collection process.

We have added in further information about the topics in the interview guide. New details are underlined:

In alignment with the aims of the project, stakeholders were asked to reflect on and respond to the concept of a ‘universal precautions’ approach to stigma reduction. This included topics such as the acceptability of the term, the feasibility of developing, designing and implementing a universal framework to stigma reduction and measuring change. They were asked to identify existing structures, policies, procedures, and frameworks where there is capacity to embed stigma reduction interventions.

• The pragmatic approach adopted for data analysis is considered well-justified.

Thank you

Results and Discussion Section

• The Results section lacks a description pertaining to financing and societal pressures. The authors should reflect on these constructs in the discussion. If data related to these constructs did not emerge during the interviews, it is important to reflect on it.

We note a typo on “societal pressure” (which was incorrectly labelled as “social pressures). There are two paragraphs in the discussion reflecting on CFIR constructs which were not apparent in the data (notably societal pressure and market pressure). We have added in comment on other Outer Setting constructs which were not apparent in the data analysed here.

• Considering the migration rate in Australia, integrating stigma reduction strategies at the structural level aimed at improving healthcare quality for Culturally and Linguistically Diverse (CALD) communities within the discussion section would substantially enhance the manuscript’s value and relevance.

While we agree with the reviewer regarding the need for better quality (and less stigmatising care) for CALD communities, we are approaching this project with the goal of improving care for all people. It could be argued that many groups (including First Nation peoples) could benefit from stigma free care, so we have chosen not to highlight any one particular group or identity.

Reviewer #2: This qualitative study evaluates how stigma reduction can be embedded within existing healthcare quality and governance structures. Based on interviews with 20 key stakeholders in the Australian BBV/STI sector and guided by the CFIR “outer setting”, the manuscript identifies accreditation, quality standards, performance pressures, and workplace legislation as structural levers for sustainable stigma reduction. The paper argues that aligning stigma reduction with quality care mechanisms suggests a more effective alternative to individual-level interventions.

This manuscript provides a timely and persuasive framing of stigma as a quality-of-care issue and makes a strong case for focusing on structural rather than purely individual-level responses. The use of CFIR, particularly the emphasis on “outer setting” mechanisms, is appropriate and clearly supports the analysis. The study draws on rich qualitative data from highly experienced stakeholders, and the findings are clearly relevant to policy and practice, especially in relation to accreditation and governance processes within healthcare systems.

Thank you for these positive comments.

However, the absence of lived-experience perspectives limits the breadth of insight, and generalisation to other areas of healthcare should be made cautiously.

The limitations section included comment that additional work is needed with consumers of health services to further explore perspectives of lived/living experience.

In addition, some recommendations remain at a relatively high policy level, with less attention to how they might be translated into everyday practice or how risks of superficial, “tick-box” implementation could be addressed.

We agree. But these translations into everyday practice are beyond the scope of this paper. We included a specific focus on “critical perspectives” in the analysis method to interrogate the potential limitations of a structural approach.

The manuscript would also benefit from light editing to simplify some of the longer and denser sentences to improve readability.

Thank you – we have undertaken a thorough edit.

Reviewer #3: This manuscript, exploring structural level mechanisms to address stigma, is about an important subject from an interesting perspective. This qualitative study has several strengths, but the manuscript also shows some weaknesses that I will try to point out below. I believe that a careful rewrite of some parts of the manuscript will improve its quality.

My comments:

1. The introduction, methods and discussion sessions contain a lot of, but important, text. For me, it was sometimes a little difficult to navigate the text. Consider adding subheadings to make it easier for the reader.

Thank you for the prompt. We have reduced the complexity and the length of the introduction to improve readability.

2. Please, review the language throughout the manuscript. During my reading, I found sentences that were incomplete (eg. Page 5, line 84 and page 11, lines 211-212), very long (e.g. page 14, lines 299-305) and xxx (e.g. xxx).

Thank you for those examples. We have reviewed and edited the paper for readability.

3. The methods section needs more information about how the study was conducted. Consider using

Attachment Submitted filename: Response to reviewers 23 02 26.docx

# Peer review: Decision Letter 1

**DOI:** 10.1371/journal.pone.0357633.r003

**Authors:** Helen Howard

18 Jun 2026

Dear Dr. Treloar,

Thank you for submitting your manuscript to PLOS ONE. After careful consideration, we feel that it has merit but does not fully meet PLOS ONE’s publication criteria as it currently stands. Therefore, we invite you to submit a revised version of the manuscript that addresses the points raised during the review process.

Please submit your revised manuscript by Aug 02 2026 11:59PM. If you will need more time than this to complete your revisions, please reply to this message or contact the journal office at plosone@plos.org. When you're ready to submit your revision, log on to https://www.editorialmanager.com/pone/ and select the 'Submissions Needing Revision' folder to locate your manuscript file.

- A letter that responds to each point raised by the academic editor and reviewer(s). You should upload this letter as a separate file labeled 'Response to Reviewers'.
- A marked-up copy of your manuscript that highlights changes made to the original version. You should upload this as a separate file labeled 'Revised Manuscript with Track Changes'.
- An unmarked version of your revised paper without tracked changes. You should upload this as a separate file labeled 'Manuscript'.

If you would like to make changes to your financial disclosure, please include your updated statement in your cover letter. Guidelines for resubmitting your figure files are available below the reviewer comments at the end of this letter.

If applicable, we recommend that you deposit your laboratory protocols in protocols.io to enhance the reproducibility of your results. Protocols.io assigns your protocol its own identifier (DOI) so that it can be cited independently in the future. For instructions see: https://journals.plos.org/plosone/s/submission-guidelines#loc-laboratory-protocols. Additionally, PLOS ONE offers an option for publishing peer-reviewed Lab Protocol articles, which describe protocols hosted on protocols.io. Read more information on sharing protocols at https://plos.org/protocols?utm_medium=editorial-email&utm_source=authorletters&utm_campaign=protocols.

As the corresponding author, your ORCID iD is verified in the submission system and will appear in the published article. PLOS supports the use of ORCID, and we encourage all coauthors to register for an ORCID iD and use it as well. Please encourage your coauthors to verify their ORCID iD within the submission system before final acceptance, as unverified ORCID iDs will not appear in the published article. *Only*  the individual author can complete the verification step; PLOS staff *cannot*  verify ORCID iDs on behalf of authors.

We look forward to receiving your revised manuscript.

Kind regards,

Helen Howard

Staff Editor

PLOS One

Journal Requirements:

If the reviewer comments include a recommendation to cite specific previously published works, please review and evaluate these publications to determine whether they are relevant and should be cited. There is no requirement to cite these works unless the editor has indicated otherwise.

[Note: HTML markup is below. Please do not edit.]

Reviewers' comments:

Reviewer's Responses to Questions

**Comments to the Author**

Reviewer #2: (No Response)

**********

2. Is the manuscript technically sound, and do the data support the conclusions?

Reviewer #2: Partly

**********

3. Has the statistical analysis been performed appropriately and rigorously?

Reviewer #2: N/A

**********

4. Have the authors made all data underlying the findings in their manuscript fully available?

The PLOS Data policy

Reviewer #2: No

**********

5. Is the manuscript presented in an intelligible fashion and written in standard English?

Reviewer #2: Yes

**********

Reviewer #2: The revised version is improved, particularly in the introduction and methods, and the authors have responded constructively to reviewer comments. The use of CFIR “outer setting” constructs provides a clear organizing framework for the analysis, and the findings are presented in a way that is meaningful for policy and practice. The discussion is also appropriately reflective about the promise and limitations of structural approaches.

I remain broadly positive about the manuscript, and I think it is close to publishable form. My remaining comments are relatively minor:

Analytic clarity could still be strengthened slightly. While the authors have improved the methods section, the paper would benefit from one or two more sentences clarifying how structural stigma was identified in the data and how inductive insights were linked to the CFIR framework.

The absence of lived-experience perspectives remains an important limitation. This is acknowledged, which is appreciated, but I encourage the authors to state even more clearly that stakeholder perspectives on structural reform cannot substitute for the perspectives of people directly experiencing stigma in healthcare.

There is a small risk of overstatement in some parts of the discussion. The argument is persuasive, but the findings are based on a relatively focused Australian BBV/STI stakeholder sample. Some phrasing could be softened slightly to emphasize that the paper identifies promising structural pathways rather than demonstrating their effectiveness.

The discussion of implementation challenges could be sharpened. The manuscript notes the possibility that accreditation and governance processes may become “tick-box” exercises. This is an important point and deserves just a little more emphasis, given that it is central to the paper’s argument.

Overall, this is a strong and worthwhile manuscript. It offers a valuable conceptual and policy contribution and, in my view, would be suitable for publication after minor final refinements to clarity, caution in claims, and emphasis on the study’s limitations.

**********

what does this mean?). If published, this will include your full peer review and any attached files.

If you choose “no”, your identity will remain anonymous but your review may still be made public.

**Do you want your identity to be public for this peer review?**  For information about this choice, including consent withdrawal, please see our Privacy Policy

Reviewer #2: No

**********

[NOTE: If reviewer comments were submitted as an attachment file, they will be attached to this email and accessible via the submission site. Please log into your account, locate the manuscript record, and check for the action link "View Attachments". If this link does not appear, there are no attachment files.]

To ensure your figures meet our technical requirements, please review our figure guidelines: https://journals.plos.org/plosone/s/figures

You may also use PLOS’s free figure tool, NAAS, to help you prepare publication quality figures: https://journals.plos.org/plosone/s/figures#loc-tools-for-figure-preparation.

NAAS will assess whether your figures meet our technical requirements by comparing each figure against our figure specifications.

# Author comment: Author response to Decision Letter 2

**DOI:** 10.1371/journal.pone.0357633.r004

26 Jun 2026

Reviewer #2:

Comment:

The revised version is improved, particularly in the introduction and methods, and the authors have responded constructively to reviewer comments. The use of CFIR “outer setting” constructs provides a clear organizing framework for the analysis, and the findings are presented in a way that is meaningful for policy and practice. The discussion is also appropriately reflective about the promise and limitations of structural approaches.

I remain broadly positive about the manuscript, and I think it is close to publishable form. My remaining comments are relatively minor:

Response:

We thank the reviewer for their comments and support of our paper.

Comment:

Analytic clarity could still be strengthened slightly. While the authors have improved the methods section, the paper would benefit from one or two more sentences clarifying how structural stigma was identified in the data and how inductive insights were linked to the CFIR framework.

Response

We have added additional text to clarify how we identify structural stigma in the data.

Our understanding of structural stigma in the BBV/STI sector is informed by our previous research speaking to governance processes and accreditation (12) as well as frameworks for understanding and responding to structural stigma in mental health (11).

The following paragraph in the methods section details how inductive insights were linked to the CFIR framework. Accreditation to quality standards is used as one example.

Analysis was guided by the Consolidated Framework for Implementation Research (CFIR) (32) which is a highly cited and widely used conceptual tool for explaining barriers and facilitators to implementation effectiveness (32). The use of CFIR prior to implementation of a program is uncommon (33) but useful to identify factors that can inform development and roll-out of future interventions (34). While activities or mechanisms to support stigma reduction might operate within a healthcare organisation (the “inner setting” as considered by CFIR), we looked for mechanisms that linked to or were determined governing processes (“outer setting”), such as those emerging from the state more broadly. Accreditation to quality standards is one example of a mechanism in the “outer setting” that has been highlighted in the literature on responses to structural stigma (11). We used constructs of the CFIR “outer setting” to categorise other activities and their governance processes that were identified or alluded to by participants (see Table 1 for definitions). In inductive analysis, we examined how participants perceived the utility and application of these mechanisms to support stigma reduction interventions, particularly identifying where participants were critical or sceptical of these aspects of the “outer setting”.

Comment:

The absence of lived-experience perspectives remains an important limitation. This is acknowledged, which is appreciated, but I encourage the authors to state even more clearly that stakeholder perspectives on structural reform cannot substitute for the perspectives of people directly experiencing stigma in healthcare.

Response:

We agree and have added this to the discussion/limitations paragraph:

The analysis presented here cannot substitute for the perspectives of people with direct experience of stigma in healthcare.

Comment:

There is a small risk of overstatement in some parts of the discussion. The argument is persuasive, but the findings are based on a relatively focused Australian BBV/STI stakeholder sample. Some phrasing could be softened slightly to emphasize that the paper identifies promising structural pathways rather than demonstrating their effectiveness.

Response:

We agree that there are additional places that we could add some qualifiers and have done so eg (new wording underlined)

In this paper, we examined the perceptions of experienced health workers in relation to possibilities that structural-level governing mechanisms that could be “put to use” and…

Participant discussions of accreditation focused on its potential usefulness to focus the attention of health executives to the matter as an External Pressure.

This is a promising step forward and might be useful to direct providers’ attention

This analysis shows that there may be potential to develop sustained and integrated approaches

We point also to existing wording that aimed to present a careful interpretation eg:

Participants’ accounts also raise the possibility of using other more general industrial relations laws

This paper was aimed at illuminating the possibilities for connecting, or creating strategic collusion between

Accrediting processes for healthcare organisations and health workers are a fertile area for further investigation along

Comment:

The discussion of implementation challenges could be sharpened. The manuscript notes the possibility that accreditation and governance processes may become “tick-box” exercises. This is an important point and deserves just a little more emphasis, given that it is central to the paper’s argument.

Response:

We agree.

We have added this sentence to the first paragraph of the discussion:

Participants also note that while there may be possibility of using these structural supports, there is enduring challenge for genuine implementation beyond “tick and flick” pro forma responses.

And added this detail (in underline) to the conclusion paragraph:

How stigma should be written into these processes, the challenges of implementing structural approaches for real effect and what consumers want from them are areas still to be examined.

Comment:

Overall, this is a strong and worthwhile manuscript. It offers a valuable conceptual and policy contribution and, in my view, would be suitable for publication after minor final refinements to clarity, caution in claims, and emphasis on the study’s limitations.

Response:

Thank you again for helping us strengthen this paper.

Attachment Submitted filename: response to reviewers 2 20 26 26.docx

# Peer review: Decision Letter 2

**DOI:** 10.1371/journal.pone.0357633.r005

**Authors:** Saima Aleem

12 Jul 2026

Dear Dr. Treloar,

Thank you for submitting your manuscript to PLOS ONE. After careful consideration, we feel that it has merit but does not fully meet PLOS ONE’s publication criteria as it currently stands. Therefore, we invite you to submit a revised version of the manuscript that addresses the points raised during the review process.

Please submit your revised manuscript by Aug 26 2026 11:59PM. If you will need more time than this to complete your revisions, please reply to this message or contact the journal office at plosone@plos.org. When you're ready to submit your revision, log on to https://www.editorialmanager.com/pone/ and select the 'Submissions Needing Revision' folder to locate your manuscript file.

- A letter that responds to each point raised by the academic editor and reviewer(s). You should upload this letter as a separate file labeled 'Response to Reviewers'.
- A marked-up copy of your manuscript that highlights changes made to the original version. You should upload this as a separate file labeled 'Revised Manuscript with Track Changes'.
- An unmarked version of your revised paper without tracked changes. You should upload this as a separate file labeled 'Manuscript'.

If you would like to make changes to your financial disclosure, please include your updated statement in your cover letter. Guidelines for resubmitting your figure files are available below the reviewer comments at the end of this letter.

If applicable, we recommend that you deposit your laboratory protocols in protocols.io to enhance the reproducibility of your results. Protocols.io assigns your protocol its own identifier (DOI) so that it can be cited independently in the future. For instructions see: https://journals.plos.org/plosone/s/submission-guidelines#loc-laboratory-protocols. Additionally, PLOS ONE offers an option for publishing peer-reviewed Lab Protocol articles, which describe protocols hosted on protocols.io. Read more information on sharing protocols at https://plos.org/protocols?utm_medium=editorial-email&utm_source=authorletters&utm_campaign=protocols.

As the corresponding author, your ORCID iD is verified in the submission system and will appear in the published article. PLOS supports the use of ORCID, and we encourage all coauthors to register for an ORCID iD and use it as well. Please encourage your coauthors to verify their ORCID iD within the submission system before final acceptance, as unverified ORCID iDs will not appear in the published article. *Only*  the individual author can complete the verification step; PLOS staff *cannot*  verify ORCID iDs on behalf of authors.

We look forward to receiving your revised manuscript.

Kind regards,

Saima Aleem

Academic Editor

PLOS One

Journal Requirements:

If the reviewer comments include a recommendation to cite specific previously published works, please review and evaluate these publications to determine whether they are relevant and should be cited. There is no requirement to cite these works unless the editor has indicated otherwise.

Please review your reference list to ensure that it is complete and correct. If you have cited papers that have been retracted, please include the rationale for doing so in the manuscript text, or remove these references and replace them with relevant current references. Any changes to the reference list should be mentioned in the rebuttal letter that accompanies your revised manuscript. If you need to cite a retracted article, indicate the article’s retracted status in the References list and also include a citation and full reference for the retraction notice.

[Note: HTML markup is below. Please do not edit.]

Reviewers' comments:

Reviewer's Responses to Questions

**Comments to the Author**

Reviewer #2: All comments have been addressed

**********

2. Is the manuscript technically sound, and do the data support the conclusions?

Reviewer #2: Yes

**********

3. Has the statistical analysis been performed appropriately and rigorously?

Reviewer #2: N/A

**********

4. Have the authors made all data underlying the findings in their manuscript fully available?

The PLOS Data policy

Reviewer #2: No

**********

5. Is the manuscript presented in an intelligible fashion and written in standard English?

Reviewer #2: Yes

**********

Reviewer #2: This is a strong and policy-relevant qualitative manuscript. The study makes a valuable contribution by reframing stigma reduction as part of quality health care and by identifying structural mechanisms, particularly accreditation and governance processes, that may be mobilized to support stigma reduction. The use of CFIR outer-setting constructs provides a coherent analytic framework, and the findings are clearly linked to implications for health-system practice.

The revisions have adequately addressed most major concerns. The methods are now clearer, the authors have acknowledged the absence of lived-experience perspectives, and the discussion has appropriately softened causal or overly strong claims. I have only minor remaining suggestions. First, the manuscript should continue to emphasize that the findings identify promising structural pathways rather than demonstrating their effectiveness. Second, the limitation regarding the absence of direct patient/consumer perspectives should remain explicit. Third, the discussion of implementation challenges, especially the risk that accreditation becomes a “tick-box” exercise, could be sharpened slightly. Finally, claims about transferability beyond the Australian BBV/STI context should be phrased cautiously.

**********

what does this mean?). If published, this will include your full peer review and any attached files.

If you choose “no”, your identity will remain anonymous but your review may still be made public.

**Do you want your identity to be public for this peer review?**  For information about this choice, including consent withdrawal, please see our Privacy Policy

Reviewer #2: No

**********

[NOTE: If reviewer comments were submitted as an attachment file, they will be attached to this email and accessible via the submission site. Please log into your account, locate the manuscript record, and check for the action link "View Attachments". If this link does not appear, there are no attachment files.]

To ensure your figures meet our technical requirements, please review our figure guidelines: https://journals.plos.org/plosone/s/figures

You may also use PLOS’s free figure tool, NAAS, to help you prepare publication quality figures: https://journals.plos.org/plosone/s/figures#loc-tools-for-figure-preparation.

NAAS will assess whether your figures meet our technical requirements by comparing each figure against our figure specifications.

# Author comment: Author response to Decision Letter 3

**DOI:** 10.1371/journal.pone.0357633.r006

13 Jul 2026

Reviewer #2: This is a strong and policy-relevant qualitative manuscript. The study makes a valuable contribution by reframing stigma reduction as part of quality health care and by identifying structural mechanisms, particularly accreditation and governance processes, that may be mobilized to support stigma reduction. The use of CFIR outer-setting constructs provides a coherent analytic framework, and the findings are clearly linked to implications for health-system practice.

The revisions have adequately addressed most major concerns. The methods are now clearer, the authors have acknowledged the absence of lived-experience perspectives, and the discussion has appropriately softened causal or overly strong claims. I have only minor remaining suggestions.

Response:

Thank for you for the support of our paper

Comment:

First, the manuscript should continue to emphasize that the findings identify promising structural pathways rather than demonstrating their effectiveness.

Response:

We note existing cautions language:

- Abstract: Innovative approaches to stigma reduction using macro, structural tools provide a promising opportunity to trial.

- Methods: more needs to be known about the feasibility of these structural mechanisms for addressing the stigmatisation of BBV/STIs and beyond.

- Results: Accreditation requirements (Policies and Laws within the “outer setting”) were seen to have the potential to drive the management team

- Results: Participants noted that regular processes of institutional management and workforce development could be adapted

- Discussion: examined the perceptions of experienced health workers in relation to possibilities that structural-level governing mechanisms

- Discussion: also raise the possibility of using other more general industrial relations laws

- Discussion: This paper was aimed at illuminating the possibilities for

We have added new wording emphasizing the possibility of these pathways:

- From line 215 - Policies and Laws as well as Performance Measurement Pressure were reflected in participant comments about the possibility of driving change by embedding stigma

- From line 322 - Within the workplace, Performance Measurement Pressure (CFIR “outer setting” construct) for individual workers was also recognised as a possible means to support stigma reduction

- From line 345 - Participant accounts support the argument for developing and testing a strategic collusion, a systematic and deliberate bringing together,

Comment:

Second, the limitation regarding the absence of direct patient/consumer perspectives should remain explicit.

Response:

We note that this limitation is already included and explicit (from line 420, in limitations section):

Additionally, while some participants would have also been consumers of health services, there is a need for future research that asks health consumers what they want to see in structural mechanisms for stigma reduction as part of quality care. The analysis presented here cannot substitute for the perspectives of people with direct experience of stigma in healthcare.

And from line 440, in concluding paragraph:

How stigma should be written into these processes, the challenges of implementing structural approaches for real effect and what consumers want from them are areas still to be examined.

Comment:

Third, the discussion of implementation challenges, especially the risk that accreditation becomes a “tick-box” exercise, could be sharpened slightly.

Response:

We have added emphasis to the concluding paragraph:

- Accrediting processes for healthcare organisations and health workers are a fertile area for further investigation including exploration of the conditions which contribute to accreditation being seen or experienced as an effective influence on healthcare organisations.

Finally, claims about transferability beyond the Australian BBV/STI context should be phrased cautiously.

Response:

There is only one mention of transferability of this analysis beyond the current context. We note existing cautious language in this section:

- In this way, features of the health system that were identified as useful for BBVs/STIs may be applicable and adaptable for other conditions, identities, and practices.

We have strengthened the language:

- Features of the health system that were identified as useful for BBVs/STIs could be explored for their applicability and adaptability for other conditions, identities, and practices.

Attachment Submitted filename: response to reviewers 3 14 07 26.docx

# Editor report: Decision Letter 3

**DOI:** 10.1371/journal.pone.0357633.r007

**Authors:** Surangi Jayakody

19 Aug 2026

Stigma reduction as quality health care: A qualitative study of structural level mechanisms to address stigma

PONE-D-25-36923R3

Dear Dr.Carla,

We’re pleased to inform you that your manuscript has been judged scientifically suitable for publication and will be formally accepted for publication once it meets all outstanding technical requirements.

Within one week, you’ll receive an e-mail detailing the required amendments. When these have been addressed, you’ll receive a formal acceptance letter and your manuscript will be scheduled for publication.

An invoice will be generated when your article is formally accepted. Please note, if your institution has a publishing partnership with PLOS and your article meets the relevant criteria, all or part of your publication costs will be covered. Please make sure your user information is up-to-date by logging into Editorial Manager at Editorial Manager® and clicking the ‘Update My Information' link at the top of the page. For questions related to billing, please contact billing support.

If your institution or institutions have a press office, please notify them about your upcoming paper to help maximize its impact. If they’ll be preparing press materials, please inform our press team as soon as possible -- no later than 48 hours after receiving the formal acceptance. Your manuscript will remain under strict press embargo until 2 pm Eastern Time on the date of publication. For more information, please contact onepress@plos.org.

Kind regards,

Surangi Jayakody, MBBS, MSc, MD

Academic Editor

PLOS One

Additional Editor Comments (optional):

Reviewers' comments:

# Editor report: Acceptance letter

**DOI:** 10.1371/journal.pone.0357633.r008

**Authors:** Surangi Jayakody

PONE-D-25-36923R3

PLOS One

Dear Dr. Treloar,

I'm pleased to inform you that your manuscript has been deemed suitable for publication in PLOS One. Congratulations! Your manuscript is now being handed over to our production team.

At this stage, our production department will prepare your paper for publication. This includes ensuring the following:

* All references, tables, and figures are properly cited

* All relevant supporting information is included in the manuscript submission,

* There are no issues that prevent the paper from being properly typeset

You will receive further instructions from the production team, including instructions on how to review your proof when it is ready. Please keep in mind that we are working through a large volume of accepted articles, so please give us a few days to review your paper and let you know the next and final steps.

Lastly, if your institution or institutions have a press office, please let them know about your upcoming paper now to help maximize its impact. If they'll be preparing press materials, please inform our press team within the next 48 hours. Your manuscript will remain under strict press embargo until 2 pm Eastern Time on the date of publication. For more information, please contact onepress@plos.org.

You will receive an invoice from PLOS for your publication fee after your manuscript has reached the completed accept phase. If you receive an email requesting payment before acceptance or for any other service, this may be a phishing scheme. Learn how to identify phishing emails and protect your accounts at https://explore.plos.org/phishing.

If we can help with anything else, please email us at customercare@plos.org.

Thank you for submitting your work to PLOS One and supporting open access.

Kind regards,

PLOS One Editorial Office Staff

on behalf of

Dr Surangi Jayakody

Academic Editor

PLOS One
